# Quantile-specific heritability of sibling leptin concentrations and its implications for gene-environment interactions

**DOI:** 10.1038/s41598-020-79116-1

**Published:** 2020-12-17

**Authors:** Paul T. Williams

**Affiliations:** Molecular Biophysics and Integrated Bioimaging, Lawrence Berkeley National Laboratory, 1 Cyclotron Road, Berkeley, CA 94720 USA

**Keywords:** Diseases, Risk factors, Genetic interaction, Heritable quantitative trait, Medical genetics

## Abstract

“Quantile-dependent expressivity” occurs when the effect size of a genetic variant depends upon whether the phenotype (e.g., leptin) is high or low relative to its distribution. Leptin concentrations are strongly related to adiposity, whose heritability is quantile dependent. Whether inheritance of leptin concentrations is quantile dependent, and whether this explains the greater heritability in women than men in accordance with their greater adiposity, and explains other gene-environment interactions, remains to be determined. Therefore, leptin and leptin receptor concentrations from 3068 siblings in 1133 sibships from the Framingham Heart Study Third Generation Cohort were analyzed. Free leptin index (FLI) was calculated as the ratio of leptin to soluble leptin receptor concentrations. Full-sib (β_FS_) regression slopes were robustly estimated by quantile regression with nonparametric significance assigned from 1000 bootstrap samples. The analyses showed β_FS_ increased significantly with increasing percentiles of the offspring’s age- and sex-adjusted leptin distribution (P_linear_ = 0.0001), which was accelerated at the higher concentrations (P_quadratic_ = 0.0003). β_FS_ at the 90th percentile (0.418 ± 0.066) was 4.7-fold greater than at the 10th percentile (0.089 ± 0.032, P_difference_ = 3.6 × 10^−6^). Consistent with quantile-dependent expressivity, the β_FS_ was greater in female sibs, which was attributable to their higher leptin concentrations. Reported gene-environment interactions involving adiposity and *LEP*, *LEPR*, *MnSOD*, *PPARγ*, *PPARγ2*, and *IRS-1* polymorphisms were consistent with quantile-dependent expressivity of leptin concentrations. β_FS_ for leptin receptor concentrations and free leptin index also increased significantly with increasing percentiles of their distributions (P_linear_ = 0.04 and P_linear_ = 8.5 × 10^−6^, respectively). In conclusion, inherited genetic and shared environmental effects on leptin concentrations were quantile dependent, which likely explains male–female differences in heritability and some gene-environment interactions.

## Introduction

Leptin is a satiety hormone that regulates body fat through hypothalamic control of energy intake and expenditure^1^. It is pro-inflammatory, pro-atherogenic and anti-apoptotic^2^. Circulating blood leptin occurs in free and protein-bound forms^3^. The soluble leptin receptor is the major leptin binding protein^4^, formed by ectodomain shedding of membrane-anchored leptin receptors^5^, whose concentrations are strongly correlated with leptin receptor cell surface expression^6^. The free leptin index (FLI, i.e., the ratio of leptin to soluble leptin receptor concentrations) is a purported measure of bioavailable leptin that can pass through the brain-blood barrier to the hypothalamus^7^. Reported heritability estimates have varied from 0.21 to 0.73 (median 0.41) for total leptin concentrations (reported *h*^*2*^: 0.21^8^, 0.32^9–11^, 0.38^12,13^, 0.39^14^, 0.42^15^, 0.45^11^, 0.46^16^, 0.59^13^, 0.60–0.65^17^, 0.63^18^, 0.73^19^); from 0.55^20^ to 0.69^21^ for the soluble leptin receptor concentrations; and 0.44–0.55 for the free leptin index^21^. Leptin concentrations are strongly skewed, and parametric tests for heritability^8,12–17,19^ and genome-wide^22,23^ and candidate gene^24^ association studies are almost always performed on logarithmically transformed leptin concentrations per their statistical requirements.

Quantile-dependent expressivity occurs when the phenotypic expression of a gene depends upon the level of the phenotype, i.e., whether the trait (e.g., leptin concentrations) is high or low relative to its distribution^25–33^. We have shown that the heritability of computed tomography (CT), dual-energy x-ray absorptiometry (DXA), and anthropometric adiposity measures are all quantile-dependent^26^, and that the effect of a 32-SNP genetic risk score (GRS) at the 90th BMI percentile was 4.2-fold greater than at the 10th percentile^25^. Circulating leptin levels exhibit a strong relationship to body fat and adipocyte cell size^34^, consistent with its function as a quantitative endocrine signal of stored fat in adipose tissue. This might predict that genetic influences on leptin concentrations are also quantile-dependent, except that: (1) residual leptin heritability persists when adjusted for adiposity^9,12^; (2) none of the non-FTO genetic loci previously associated with BMI attained < 10^−6^ significance with leptin concentrations^22^, (3) the four non-FTO loci in or near *LEP*, *SLC32A1*, *GCKR*, *CCNL1* that attained genome-wide association with leptin (*P* < 5 × 10^−8^) persisted when adjusted for BMI^22^.

An important consequence of quantile-dependent expressivity is that the selection of subjects for characteristics that distinguish high versus low phenotypes can yield different genetic effects^33^. Women secrete more leptin than men due to their larger percentage of body fat^35^, greater subcutaneous fat storage^35,36^, and low testosterone^37^. Being overweight or obese accentuates the sexual dimorphism in leptin secretion^38^. Genetic influences on leptin concentrations are also greater in women. For example, Martin et al.^39^ reported that leptin heritability was greater in women than men (*h*^*2*^ = 0.57 vs. *h*^*2*^ = 0.31) and that women’s had higher average leptin concentrations (29.34 ± 0.94 vs. 10.80 ± 0.56 ng/ml), as did Hasselbalch et al.^13^ (i.e., *h*^*2*^_female_ = 0.59 vs. *h*^*2*^_male_ = 0.38), and Rotimi et al.^14^. Kaprio et al.^11^ reported that additive genetic effects on leptin concentrations were five-fold larger in women than men in accordance with their higher average leptin concentration (16.8 ± 9.5 vs. 6.4 ± 3.5 ng/ml, *P* < 0.0001). Moreover, the effect of *PPARγ2 (*peroxisome proliferator-activated receptor γ2) rs1801282 genotypes on leptin concentrations is significantly greater in women than men^40^. In addition to sex, adiposity^41–47^, diet^48^, and smoking^49^ are reported to modify the effects of genes on leptin concentrations.

We therefore sought to test whether shared environmental and inherited factors affecting leptin concentrations in sibs were quantile-dependent in a large population cohort (Framingham Heart Study^50^). Untransformed concentrations were analyzed because quantile regression does not require normality^51,52^, and no biological justification has yet been given for its logarithmic transformation. We also re-analyze published studies of leptin that measured genetic variants directly from the perspective of quantile-dependent expressivity. The results suggest that quantile-dependent expressivity: (1) provides a simple explanation for the greater leptin heritability in women than men^11,13,14,39,40^ and (2) is consistent with the genotype-specific effects of weight^41–47^, diet^48^, and smoking^49^ on leptin concentrations.

## Methods

The methods have been described previously^25–33^, but are repeated here for completeness. The data were obtained from the National Institutes of Health FRAMCOHORT, GEN3, FRAMOFFSPRING Research Materials obtained from the National Heart Lung and Blood Institute (NHLBI) Biologic Specimen and Data Repository Information Coordinating Center. Subjects were at least 16 years of age and not self-identified as nonwhite or Hispanic. Leptin and soluble leptin receptor concentrations were measured on stored EDTA plasma samples frozen at -80 °C from the first examination of the Framingham Third Generation Cohort^50^ by ELISA (R&D Systems Inc., Minneapolis, MN) with an average interassay coefficients of variation < 5%^53^. Free leptin index, a purported measure of bioavailable leptin not bound to its soluble receptor, was calculated as the ratio of leptin to leptin-receptor concentrations. Subjects used in the current analyses were at least 16 years of age, were not taking medications for diabetes, and were self-identified as non-Hispanic white. These analyses were approved by Lawrence Berkeley National Laboratory Human Subjects Committee (HSC) for protocol “Gene-environment interaction vs quantile-dependent penetrance of established SNPs (107H021)” Approval number: 107H021-13MR20. LBNL holds the Office of Human Research Protections Federal wide Assurance number FWA 00006253. All surveys were conducted under the direction of the Framingham Heart Study human use committee guidelines, with signed informed consent from all participants or parent and/or legal guardian if < 18 years of age.

### Statistics

Age and sex adjustment was performed using standard least-squares regression with the following independent variables: female (0,1), age, age^2^, female x age, and female x age^2^. Full-sibling correlations and regression coefficients (β_FS_) were obtained by constructing all possible pairs using double entry^54^, with an adjusted Σ(k_i_ − 1) degrees of freedom, where k_i_ is the number of offspring in family i and the summation is taken over all i, i = 1,…, N nuclear families.

**Figure 1:**
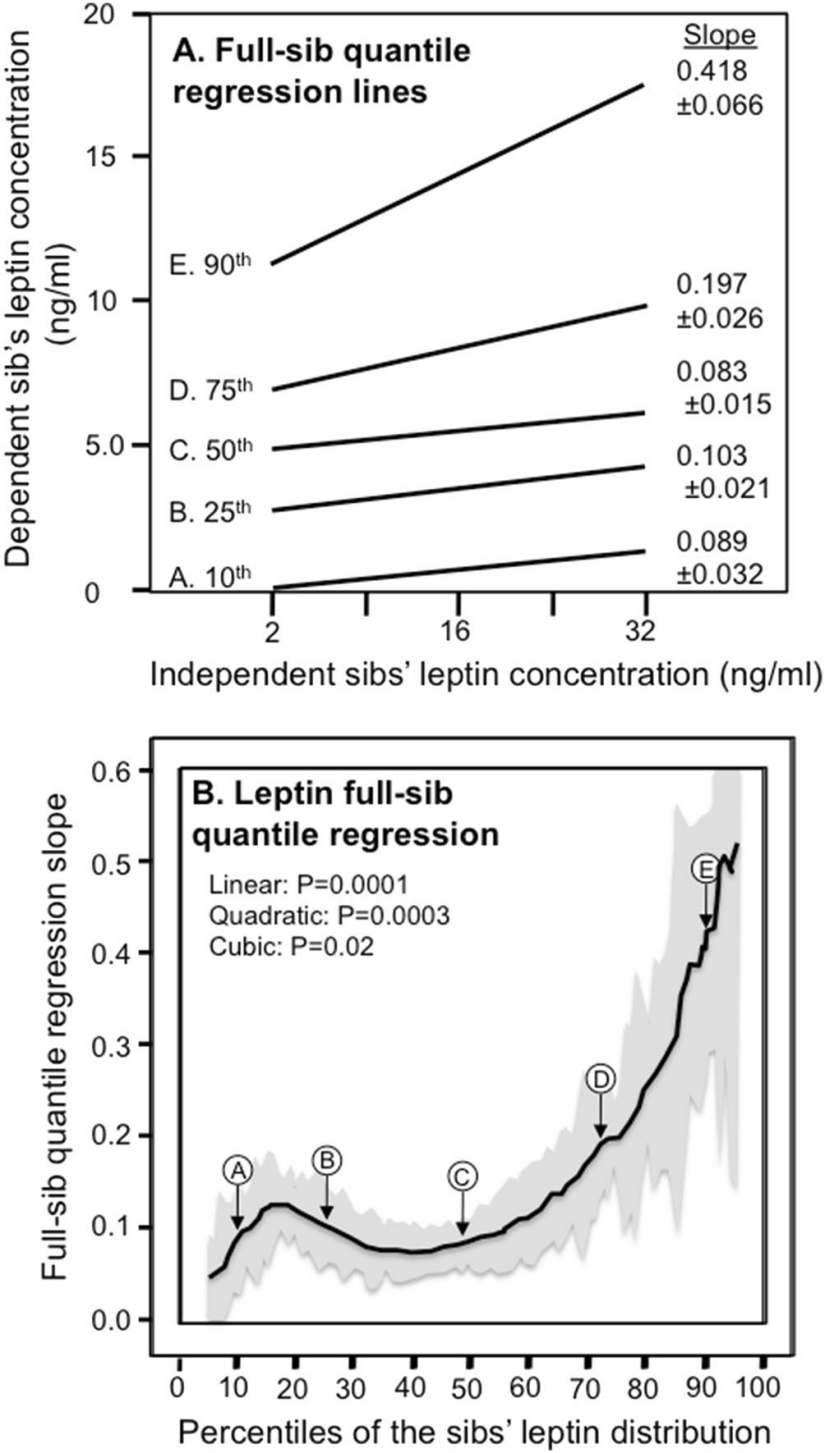
(**A**) Full sib regression slopes (β_FS_) for selected quantiles of the leptin concentrations from 3068 siblings in 1133 sibships. The slopes became progressively greater (i.e., steeper) at higher quantiles of the leptin distribution (i.e., > 50th percentile). (**B**) The selected quantile-specific regression slopes from the upper panel were included with those of other quantiles to create the quantile-specific β_FS_ function. Significance of the linear, quadratic and cubic trends and the 95% confidence intervals (shaded region) determined by 1000 bootstrap samples.

Simultaneous quantile regression was performed using the “sqreg” command of Stata (version. 11, StataCorp, College Station, TX) with one thousand bootstrap samples drawn to estimate the variance–covariance matrix for the 91 quantile regression coefficients (β_FS_) between the 5th and 95th percentiles, and the post-estimation procedures (test and lincom) to test linear combinations of the β_FS_ slopes after estimation with Σ(k_i_-1) degrees of freedom. Quantile-specific sib-sib concordance was assessed by: (1) estimating quantile-specific β_FS_-coefficient for the 5th, 6th, …, 95th percentiles of the sample distribution using simultaneous quantile regression (Fig. 1, the < 5th and > 95th percentiles ignored because they were thought to be less stable); (2) plotting the quantile-specific β_FS_ coefficients versus the percentile of the trait distribution; and (3) testing whether the resulting graph is constant, or changes as a linear, quadratic, or cubic function of the percentile of the trait distribution using orthogonal polynomials^55^. Female β_FS_ slopes refer to all sib-pairs where a female sib is the dependent variable and male or female sibs are the independent variable, male β_FS_ slopes refer to all sib-pairs where a male sib is the dependent variable and male or female sibs are the independent variable. Unadjusted regression slope refer to an unadjusted sib value as the dependent variable versus the adjusted remaining sib values as the independent variables. Slopes are presented ± SE.

**Figure 2:**
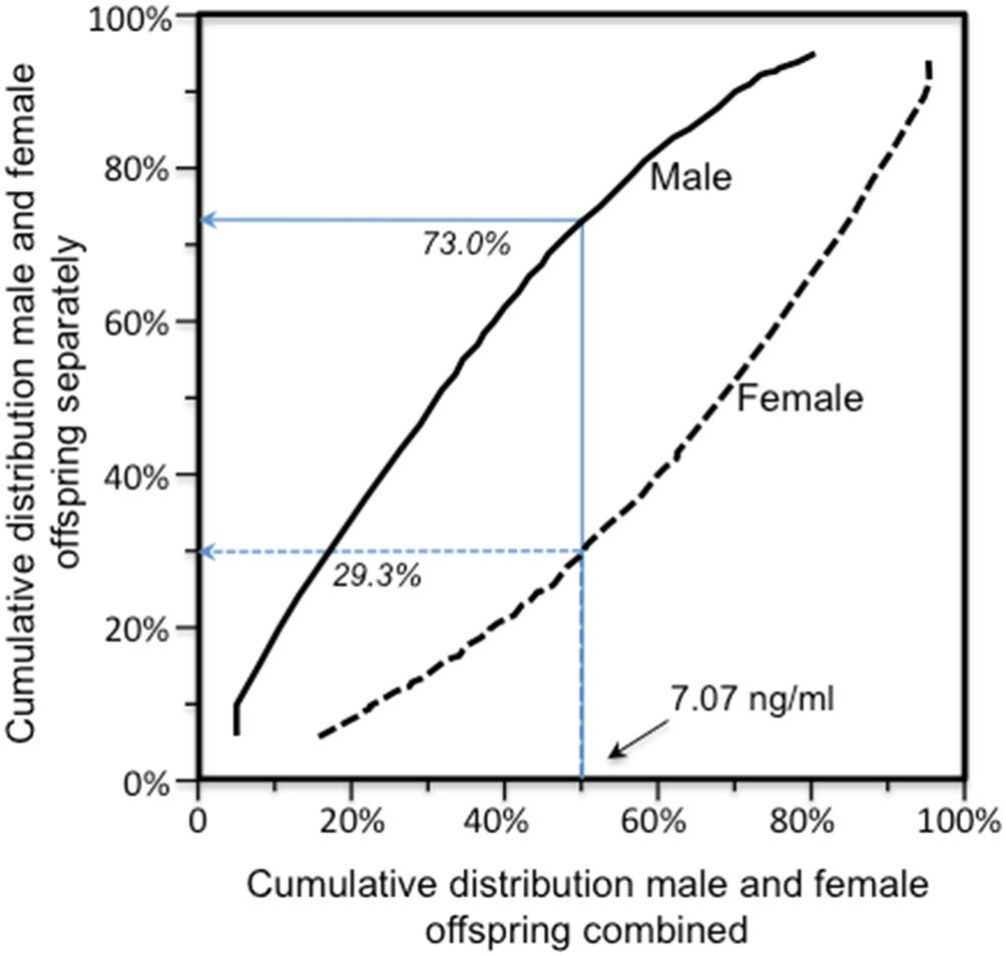
Probability-probability (*P*–*P*) plot of the distribution of leptin concentrations in males and females separately (vertical axis) versus the sexes combined, showing the percentiles of the male and female distribution having the same leptin concentration (see methods).

When β_FS_ for male and female sib are compared on the same graph, their quantile-specific functions compare their slopes at the corresponding percentiles of their separate distribution (e.g., the slope at the 50th percentile of the females’ distribution versus the slope at the 50th percentile of the males’ distribution). However, the leptin concentration at the 50th percentile of the females’ distribution will be greater then the 50th percentile of the males’ distribution. Quantile-specific expressivity postulates that the genetic effects depend upon the leptin concentration. Therefore, additional displays were created based on probability-probability plots (*P*–*P* plots, Fig. 2)^56^ that re-plot the males’ and females β_FS_ at the same leptin concentration. For example, Fig. 2 shows that the 50th percentile of the leptin distribution for male and female offspring combined was 7.07 ng/ml (horizontal axis). This corresponds to the 29.3^rd^ percentile of the female distribution and 73^rd^ percentile of the male distribution (vertical axis). Thus plotting the β_FS_ at the females’ 29.3th percentile and males’ 73^rd^ percentile at the 50th percentile of their combined distribution results in their β_FS_’s being compared at the same leptin concentration. This process was repeated for each percentile of their combined distribution (interpolated where required) to compare male and female β_FS_ when matched by leptin concentrations.

## Results

Women and men were of similar age {female vs. male mean (SD): 39.9 (8.7) vs. 40.4 (8.6) years}. Compared to men, women had higher average leptin (17.95 (16.96) vs. 8.08 (6.04) ng/ml) and soluble leptin receptor concentrations {20.03 (8.91) vs. 18.91 (8.16) ng/ml} and higher free leptin index {1.21 (1.55) vs. 0.42 (5.16)} but lower BMI {26.0 (6.1) vs. 28.0 (4.7) kg/m^2^}. As expected, BMI correlated positively with leptin concentrations (r = 0.76) and the free leptin index (r = 0.64), and negatively with soluble leptin receptor concentrations (r = -0.27) when age and sex adjusted.

### Traditional estimates of familial concordance and heritability

There were 3068 full-sibs in 1133 sibships with age and sex-adjusted leptin and soluble leptin receptor concentrations, whose traditional least-squares full-sib regression slopes (β_FS_) were 0.17 ± 0.02 (*P* = 1.8 × 10^−14^) for leptin and 0.28 ± 0.02 (*P* < 10^−6^) for soluble leptin receptor concentrations, and 0.18 ± 0.02 (*P* = 2.2 × 10^−15^) for the free leptin index. If dominance, common environment, and assortative mating effects are negligible, then these slopes would correspond to heritability estimates of 0.34 ± 0.04 for serum leptin concentrations, 0.56 ± 0.04 for soluble leptin receptor concentrations, and 0.36 ± 0.04 for the free leptin index^57^.

### Quantile-dependent expressivity

Figure 1A presents the full-sib regression slopes (β_FS_) at the 10th, 25th, 50th, 75th, and 90th percentiles of the sibs’ age- and sex-adjusted leptin distribution. The slopes get progressively steeper with increasing percentiles of the distribution. β_FS_ at the 90th percentile was 4.7-fold greater than at the 10th percentile (P_difference_ = 3.6 × 10^−6^). These slopes, along with those of the other percentiles between the 5th and 95th percentiles, are presented in the quantile-specific β_FS_ plot in Fig. 1B. They show β_FS_ increased with increasing percentiles of the offspring’s distribution (i.e., slope ± SE increased 0.0034 ± 0.0009 per percentile, P_linear_ = 0.0001) and that the increase accelerated at higher concentrations (P_quadratic_ = 0.0003). Quantile-specific β_FS_ was significant (*P* ≤ 0.005) for all individual percentiles between the 10th and 94th percentiles of the sibs’ leptin distribution. If β_FS_ was the same over all quantiles as traditionally assumed, then the line segments in Fig. 1A would be parallel, and the graph in Fig. 1B would show a flat line having zero slope.

**Figure 3:**
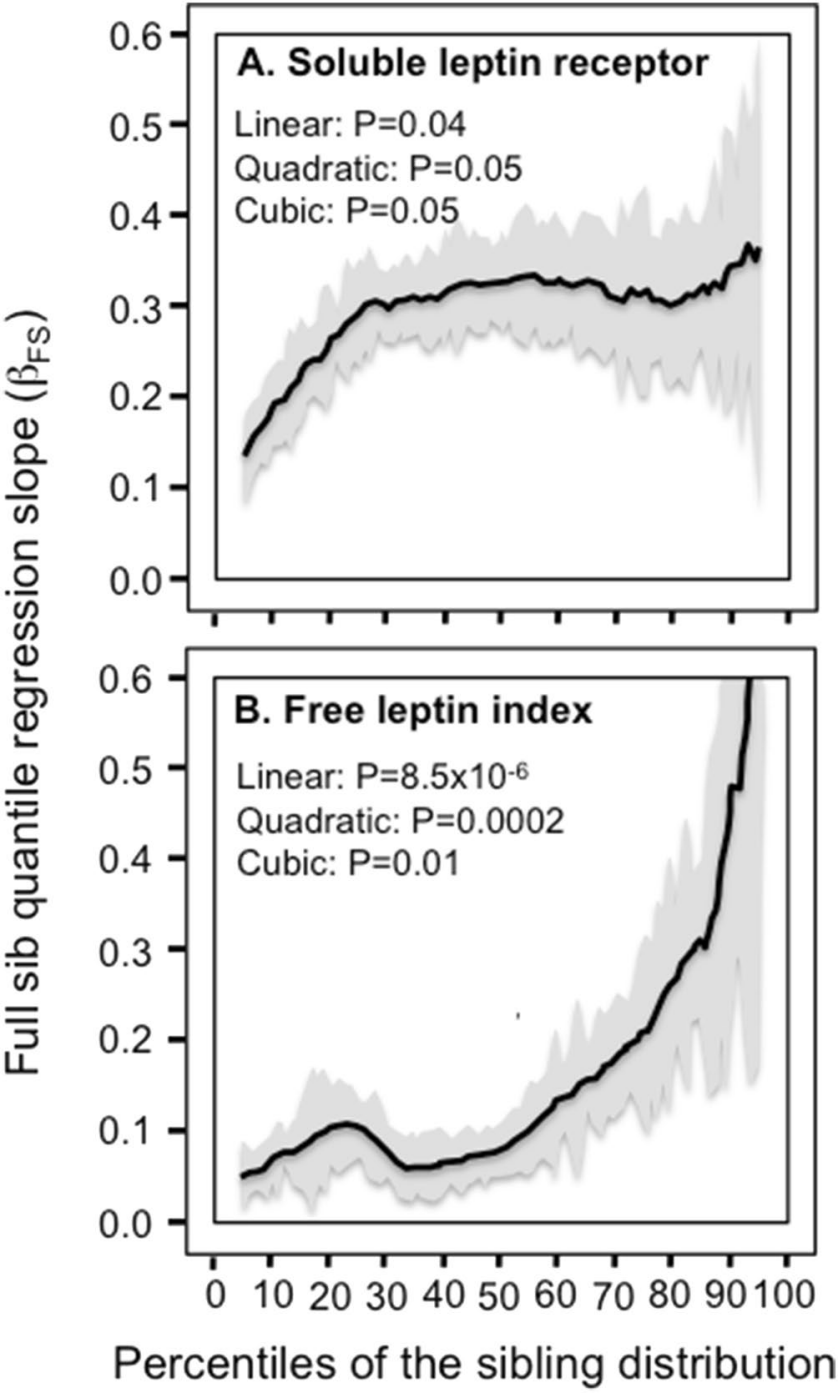
Quantile-specific full sib regression slopes (β_FS_) by quantiles of the: A) soluble leptin receptor distribution; B) free leptin index (FLI, i.e., the ratio of leptin to soluble leptin receptor concentrations).

Figure 3 show significant quantile-dependent increases in the slopes for the soluble leptin receptor concentrations and the free leptin index, i.e., each one-percent increase in the phenotype distribution increased β_FS_ by 0.0014 ± 0.0007 (P_linear_ = 0.04) for leptin receptor concentrations, and by 0.0043 ± 0.0010 (P_linear_ = 8.5 × 10^−6^) for the free leptin index. The increases were nonlinear for both the leptin receptor and the free leptin index (i.e., significant convexity for leptin index and significant concavity for the leptin receptor, with some cubic effects).

### Male–female differences in heritability

**Figure 4:**
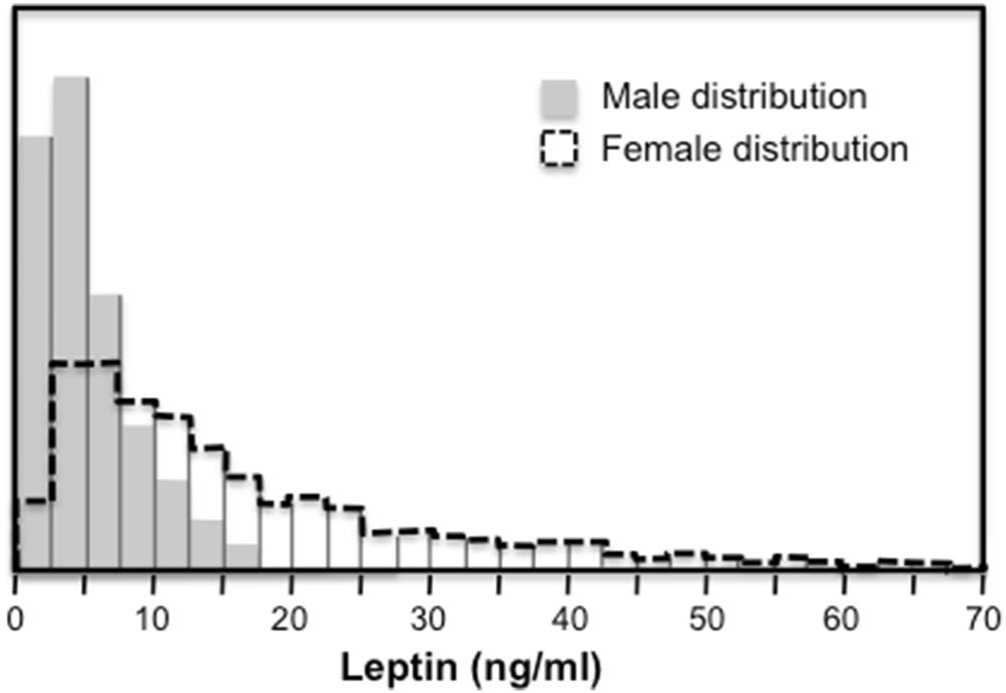
Distribution of plasma leptin concentrations in Framingham Heart Study women and men. The area under each curve represents the percentage of the distribution. For example, 10 percent of the males had leptin concentration ≤ 1.38 ng/ml, 50 percent were ≤ 4.20 ng/ml and 90 percent were ≤ 12.52 ng/ml. For women, 10 percent had leptin concentration ≤ 3.38 ng/ml, 50 percent were ≤ 11.97 ng/ml, and 90 percent were ≤ 41.10 ng/ml.

**Figure 5:**
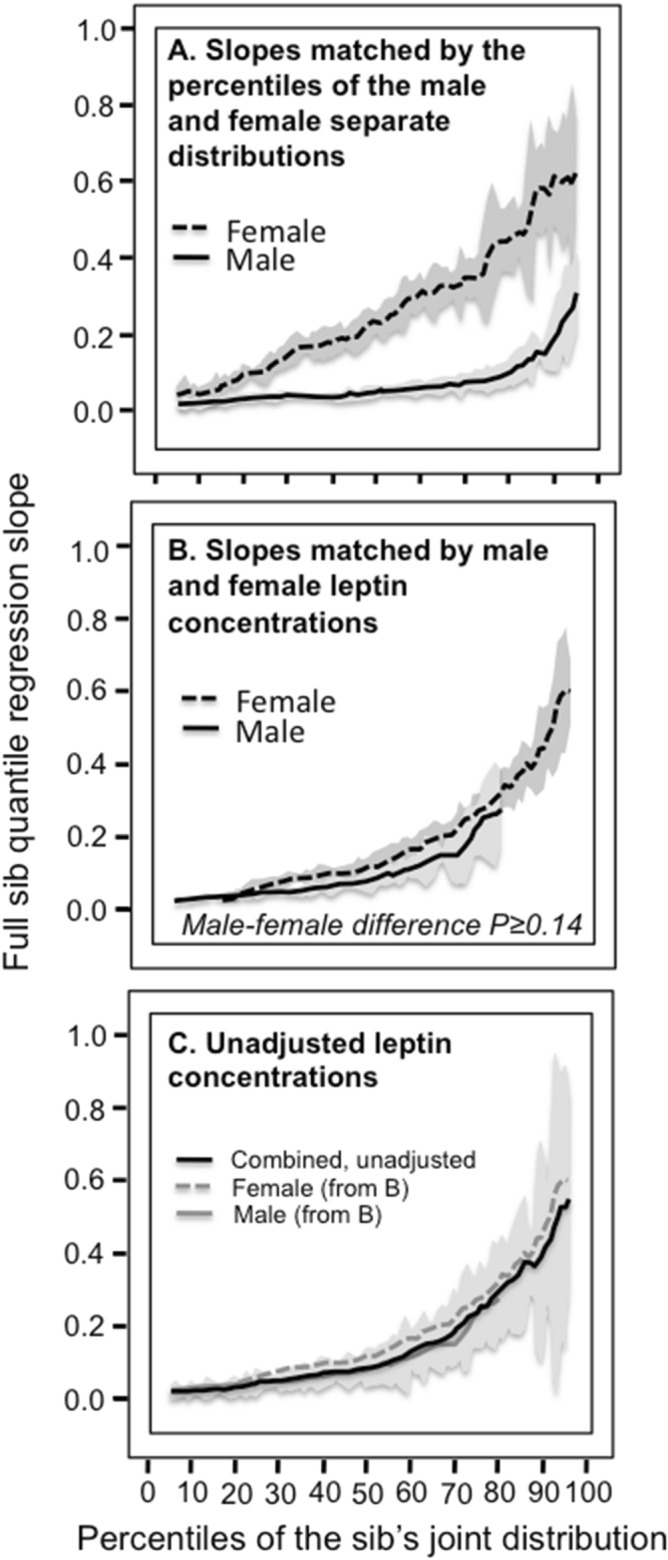
(**A**) Full-sib regression slopes (β_FS_) in male and female offspring separately from age- and sex-adjusted male and female pairs, showing their significant difference when the slopes are compared at their corresponding percentiles (the male sibs’ vs. the female sibs’ β_FS_ compared at the 5th percentile of separate distributions, the 6th percentile of their separate distributions, …, 95th percentile of their separate distributions). Shaded area designates ± SE; (**B**) Full-sib regression slopes (β_FS_) in male and female sibs showing the significant difference is eliminated when the slopes are compared at their corresponding leptin concentrations (the male sibs’ vs. the female sibs’ β_FS_ translated using probability-probability (P-P) plots to re-plot their leptin concentrations at the 5th percentile of their combined distribution, the 6th percentile of their combined distribution, …, 95th percentile of their combined distribution). Shaded area designates ± SE. (**C**) Full sib regression slopes for male and female sibs combined without adjustment for sex, showing the unadjusted analysis provides a simpler description of the quantile increase based solely on the percentiles of their unadjusted leptin concentrations. Note that the separate curves for male and female sibs fall fully within the 95% confidence interval (shaded area) for their combined sex-unadjusted analysis.

Figure 1B showed that leptin heritability increased significantly with increasing quantiles of the offspring’s leptin distribution when male and female age- and sex-adjusted sibling data were combined. However, Fig. 4 shows the leptin distribution in females is shifted towards to the right of the males’ distribution, ergo the females’ β_FS_ should be greater than that of the males. In fact, as traditionally estimated by least squares regression, leptin’s β_FS_ was higher in females than males (0.26 ± 0.03 vs. 0.07 ± 0.01 for the total sample, *P* < 10^−15^). Moreover, Fig. 5A shows that the quantile-specific β_FS_ was higher in females than males at each percentile of their respective distribution.

The problem with Fig. 5A is that comparing male and female β_FS_ at their 10th percentiles means comparing the male β_FS_ at an unadjusted leptin concentration of 1.38 ng/ml with the female β_FS_ at an unadjusted concentration of 3.38 ng/ml. At the 50th percentile, the males’ β_FS_ at 4.20 ng/ml is being compared to the female β_FS_ at 11.97 ng/ml, and at the 90th percentile, the males’ β_FS_ at 12.52 ng/ml is being compared to the females’ β_FS_ at 41.10 ng/ml. Specifically, quantile-dependent expressivity predicts an increase in genetic effects with increasing leptin concentrations. Therefore the male and female β_FS_ graphs were re-plotted to correspond to the same leptin concentrations in Fig. 5B using a probability-probability (*P*–*P*) plot (Fig. 2, see methods). The significant differences between the male and female β_FS_ plots were eliminated when matched by leptin concentrations. In fact, the relationship of β_FS_ to the percentiles of the leptin distribution was more easily described by quantile regression of the leptin concentrations unadjusted for sex in Fig. 5C. The bump below the 40th percentile of the age and sex-adjusted leptin distribution in Fig. 1B was eliminated for the unadjusted concentrations, along with the significant cubic effect (adjusted: *P* = 0.02; unadjusted *P* = 0.25).

**Figure 6:**
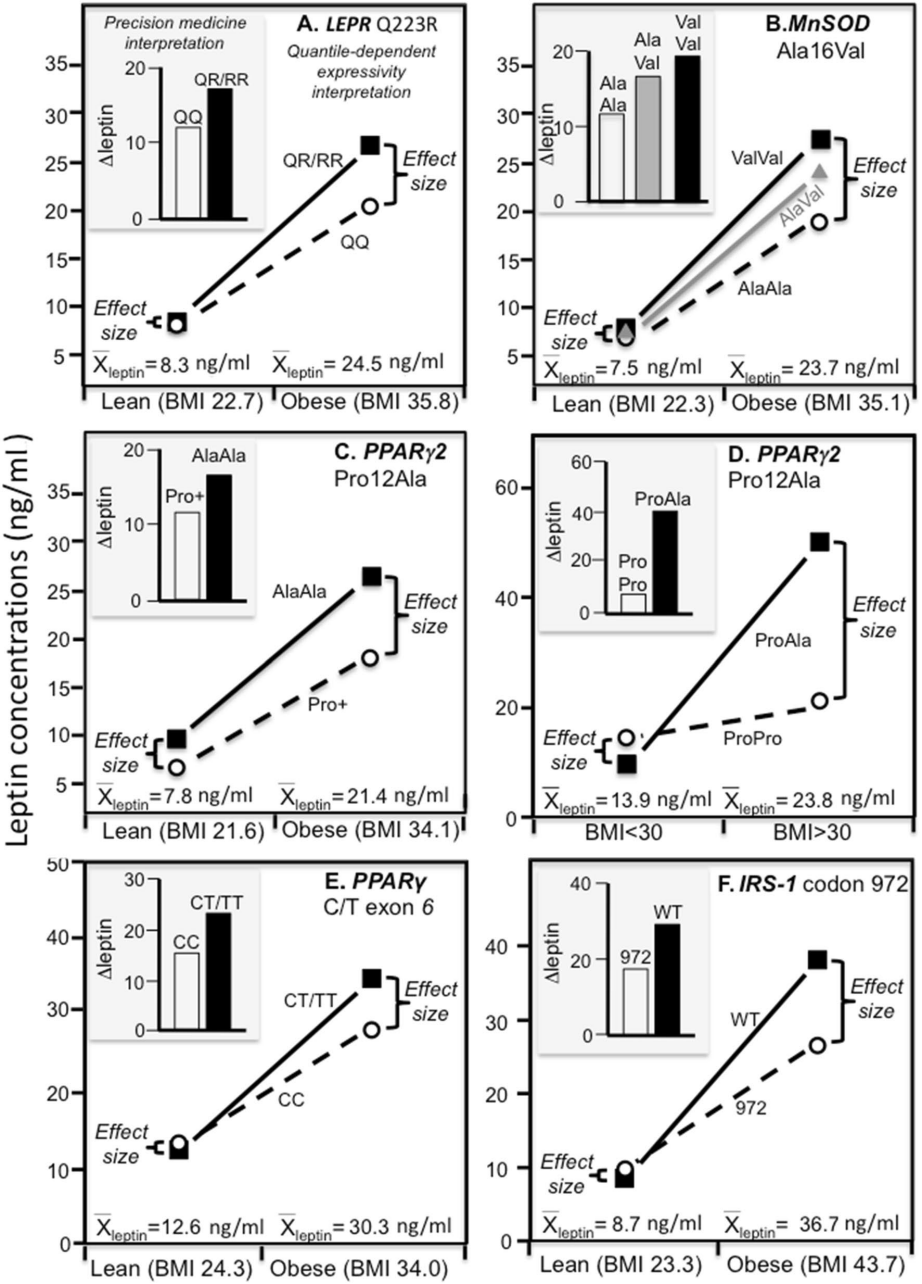
Precision medicine perspective of genotype-specific effects of obesity on leptin concentrations (histogram inserts) versus a quantile-dependent expressivity perspective (line graphs showing larger genetic effect size at higher average leptin concentrations) in: (**A**) QQ-homozygotes and R-allele carriers of the leptin receptor (*LEPR*) Q223R polymorphism (P_interaction_ = 0.05) reported by Becer et al.^42^; (**B**) AlaAla-homozygotes versus AlaVal-heterozygotes (*P* = 0.03) and ValVal-homozygotes (*P* = 0.0001) of the manganese superoxide dismutase (*MnSOD*) Ala16Val polymorphism reported by Becer et al.^43^; (**C**) AlaAla-homozygotes vs Pro-allele carriers of the peroxisome proliferator-activated receptor γ2 (*PPARγ2*) Pro12Ala polymorphisms (P_interaction_ = 0.03) reported by Becer et al.^41^; (**D**) *PPARγ2* Pro12Pro-homozygotes vs Pro12Ala T2DM patients (P_interaction_ = 0.03) reported by Simon et al.^40^; (**E**) CC-homozygotes vs T-allele carriers of the peroxisome proliferator-activated receptor γ (*PPARγ*) C/T exon 6 polymorphism (P_interaction_ = 0.03) reported by Meirhaeghe et al.^44^; (**F**) 972-variant vs wild type at codon 972 of the insulin receptor substrate-1 (IRS-1) polymorphism reported by Krempler et al.^45^.

**Figure 7:**
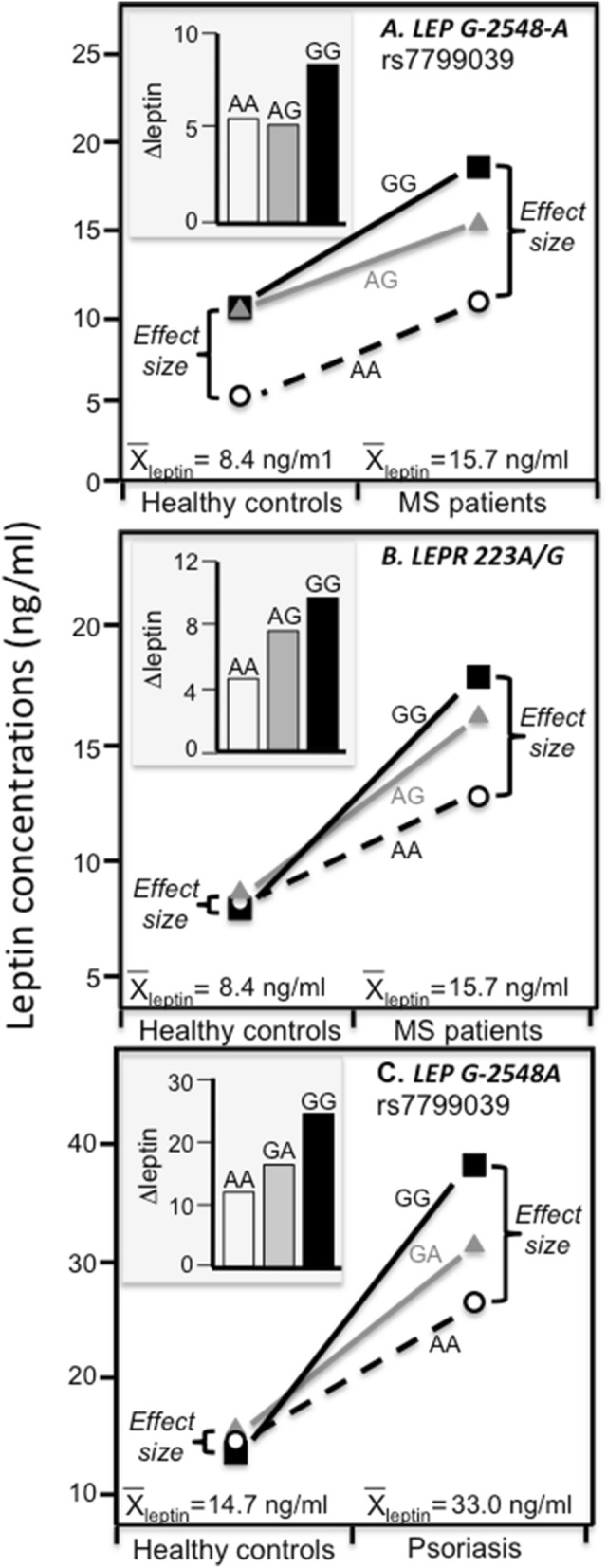
Precision medicine perspective of genotype-specific effects of multiple sclerosis (MS) and psoriasis on leptin concentrations (histogram inserts) versus a quantile-dependent expressivity perspective (line graphs showing larger genetic effect size at higher average leptin concentrations) in: (**A**) GG homozygotes than AA homozygotes (*P* = 0.01) or AG heterozygotes (*P* = 0.004) of the leptin gene (*LEP*) rs7799039 polymorphism reported by Farrokhi et al.^58^; (**B**) in AA versus AG (*P* = 0.005), AA versus GG (*P* = 3.7 × 10^−5^), and AG versus GG genotypes (*P* = 0.05) of the leptin receptor gene (LEPR) 223A/G polymorphism reported by Farrokhi et al.^58^; and (**C**) in GG homozygotes than GA (1.9 × 10^−6^) and AA genotypes (*P* < 10^−7^) of the G-2548A *LEP* polymorphism as reported by Abdel Hay et al.^60^.

The preceding analyses of β_FS_ in Framingham Study sibships lack the specificity of directly measured genotypes. This limitation may be partly addressed by reinterpreting published studies that measured genetic variants directly from the perspective of quantile-dependent expressivity (Figs. 6 and 7). Specifically, in each case, the difference in genetic effect size by environmental condition (adiposity, diet, smoking) or disease status (multiple sclerosis, systemic lupus erythematosus, psoriasis*)* corresponds to a larger genetic effect for the higher average leptin concentration, i.e., quantile-dependent expressivity.

### Adiposity

Becer et al. reported on the modifying effects of *LEPR* rs1137101^42^, *MnSOD* rs4880^43^, and *PPARγ2* rs1801282 polymorphisms^41^ on leptin concentrations in obese and non-obese subjects. The histograms of Fig. 6A–C shows that the leptin difference between obese and non-obese patients was greater in *LEPR* R-allele carriers than QQ homozygotes (18.2 ± 1.8 vs. 12.3 ± 2.0 ng/ml, *P* = 0.03), greater in *MnSOD* AlaVal heterozygotes (16.8 ± 1.6 ng/ml) and ValVal homozygotes (19.5 ± 1.3 ng/ml) than AlaAla homozygotes (11.8 ± 1.5 ng/ml, *P* = 0.02 and 0.0001, respectively), and greater in *PPARγ2* AlaAla homozygotes than carriers of the Pro-allele (16.8 ± 2.2 vs. 11.7 ± 0.8 ng/ml, *P* = 0.03). However, mean leptin concentrations were nearly three-fold higher in the obese. Consistent with quantile dependent expressivity, the line graphs of Fig. 6A–C show the difference between genotypes was greater at the higher mean leptin concentrations of the obese subjects than at the lower mean leptin concentrations of the non-obese subjects.

The histogram in Fig. 6D shows that the leptin difference between obese and non-obese diabetics reported by Simon et al.^40^ was greater in the Pro12Ala genotype than Pro12Pro genotype of *PPARγ2* (40.3 ± 10.7 vs. 7.2 ± 2.2 ng/ml, P_interaction_ = 0.002). The line graph shows this may be attributable to the larger genotype difference (28.4 ± 10.7 ng/ml) in obese subjects because of their higher average leptin concentrations (23.8 ± 1.9 ng/ml) vis-à-vis the smaller genotype difference (-4.7 ± 2.5 ng/ml) in non-obese subjects because of their lower average concentrations (13.9 ± 1.1 ng/ml).

Meirhaeghe et al.^44^ reported there was a significant gene-adiposity interaction (*P* < 0.03) involving the silent C → T substitution in exon 6 of the PPARγ gene. The histogram in Fig. 6E shows a greater leptin difference between obese and non-obese subjects in carriers of the T-allele than CC homozygotes (23.2 ± 3.1 vs. 15.4 ± 1.7 ng/ml). However, average leptin concentrations were greater in the obese than nonobese subjects (30.3 ± 1.4 vs. 12.6 ± 0.4 ng/ml) and, as shown in the accompanying line graph, the difference between genotypes was substantially greater in the obese (6.7 ± 3.4 vs. −1.1 ± 0.8 ng/ml).

Data reported by Krempler et al.^45^ showed that the leptin difference between obese and nonobese subjects was greater in wild type homozygotes than heterozygotes of the *IRS-1* codon 972 variant (P_interaction_ = 0.0004, Fig. 6F). However, average leptin concentrations were greater in the obese than non-obese subjects (36.7 ± 1.5 vs. 8.7 ± 0.5 ng/ml) and, as shown in the accompanying line graph, the difference between genotypes was substantially greater in the obese (11.1 ± 2.9 vs. −0.8 ± 1.7 ng/ml).

Eldosouky et al.^46^ reported significant leptin differences between genotypes in obese (*P* ≤ 0.004) but not non-obese Saudi children (*P* > 0.54) for *LEPR* Gln223Arg rs1137101 (G-carriers minus AA: 3.0 vs. −0.1 ng/ml) and *LEP* G2548A rs7799039 (GG minus A-carriers: 4.5 vs. −0.7 ng/ml) in accordance with the higher average leptin concentrations in the obese children (33.9 vs. 14.5 ng/dl, *P* < 0.001).

Another study, by Le Stunff et al.^47^, reported a greater effect of fat mass on serum leptin concentrations in obese girls who were + / + homozygotes (regression equation: leptin = 6 + 0.7kg_fat_) than −/− homozygotes (leptin = -8.3 + 1.9kg_fat_) of the *LEP* –2,549 polymorphism. The result was replicated in two separate cohorts. However, average leptin concentrations increased with increasing fat mass. There was no genotype difference for the less fat girls who had lower leptin concentration, and diverging leptin concentrations between genotypes as average leptin concentrations increased with increasing fat mass (their Figs. 2 and 3).

### Diet

Rafiee et al.^48^ reported that the leptin difference between high (≥ 54%) and low (< 54%) carbohydrate intake was greater in *Del* carriers than *Ins/Ins* homozygotes of the *APOB Ins/Del* polymorphism (9.6 ± 1.4 vs. −0.8 ± 1.3 ng/ml, P_interaction_ = 0.01). This corresponded with a larger genetic effect size on the low- than the high-carbohydrate diet (7.6 ± 1.1 vs. −2.8 ± 1.6 ng/ml) in accordance with the higher average leptin concentrations of the low carbohydrate diet (27.5 ± 0.6 vs. 24.9 ± 0.8 ng/ml).

### Smoking

Martin et al.^49^ reported a significant genotype by smoking interaction (*P* = 0.001), where leptin heritability was greater in nonsmokers than smokers (*h*^*2*^ = 0.60 vs. *h*^*2*^ = 0.45), in accordance with the higher average leptin concentrations in the nonsmokers (10.71 ± 0.26 vs. 8.16 ± 0.47 ng/ml).

### Multiple sclerosis

Data presented by Farrokhi et al.^58^ suggests that the leptin difference between multiple sclerosis (MS) patients and age-sex-matched controls (Fig. 7A histogram) was significantly greater in GG homozygotes (difference ± SE: 8.30 ± 0.69 ng/ml) than in AA homozygotes (5.49 ± 0.67, *P* = 0.003) or AG heterozygotes (5.14 ± 0.69 ng/ml, *P* = 0.001) of *LEP* rs7799039 polymorphism. The associated line graph shows that average leptin concentrations were, however, higher in the MS than matched control patients (15.70 ± 0.28 vs. 8.39 ± 0.27 ng/ml, *P* < 0.0001), and that this corresponded to greater differences between genotypes (GG minus AA: 7.81 ± 0.66 in MS vs. 5.00 ± 0.69 ng/ml in matched controls). The Fig. 7B histogram shows that MS’s and control’s leptin differences also significantly differed by the *LEPR* 223A/G polymorphism, *P* = 0.005 for AA versus AG genotypes, *P* = 3.7 × 10^−5^ for AA versus GG genotypes, and *P* = 0.05 for AG versus GG genotypes. The associated line graph shows that the effect can again be attributed to the genotype differences being greater in the MS than healthy patients in accordance with the greater mean concentrations in MS than healthy patients.

### Systemic lupus erythematosus

Afroze et al.^59^ reported that leptin concentrations were significantly higher in G-allele carriers than AA homozygotes of the *LEPR* 223A/G polymorphism (25.6 ± 1.2 vs. 16.4 ± 2.2 ng/ml, *P* < 0.001) for patients with systemic lupus erythematosus and apparently not in matched controls in accordance with the higher average leptin concentrations of the patients (23.9 ± 1.95 vs. 14.8 ± 1.04 ng/ml, *P* < 0.001).

### Psoriasis

Abdel Hay et al.^60^ reported plasma leptin concentrations differed significantly between genotypes of the G-2548A *LEP* polymorphism in 94 psoriasis patients (*P* < 0.001) but not 100 healthy controls (*P* = 0.19). Figure 7C histogram shows the leptin difference between psoriasis patients and control was significantly greater in GG homozygotes (24.4 ± 1.1 ng/ml) than both GA heterozygotes (16.3 ± 1.3, *P* = 1.9 × 10^−6^) and AA homozygotes (12.0 ± 2.1 ng/ml, *P* < 10^−7^). Quantile-dependent expressivity would attribute the genotype differences to the larger genetic effect size in psoriasis patients due to their higher average leptin concentration (mean ± SE: 33.0 ± 0.7 vs. 14.7 ± 0.34 ng/ml, *P* < 0.001), as illustrated in the line graph of Fig. 7C.

#### Log leptin

β_FS_’s for log leptin concentrations were 0.1994 ± 0.0209 for the males and females combined, showed no difference between female and male sibs (0.1875 ± 0.0368 and 0.2097 ± 0.0363), and did not increase with increasing leptin concentrations (0.0001 ± 0.0005 per percent increment, *P* = 0.88). β_FS_’s for log leptin receptor concentrations were 0.3268 ± 0.0202 for the males and females combined, were somewhat greater in females than males (0.3505 ± 0.0337 vs. 0.2996 ± 0.0371), and decreased nonlinearily with increasing log leptin receptor concentrations (P_linear_ = 3.7 × 10^−6^, P_quadratic_ = 5.3 × 10^−5^, P_cubic_ = 5.3 × 10^−5^).

## Discussion

Quantile-regression does not require normality^51,52^, and provides the opportunity to assess quantile-specific genetic effects as originally measured. This approach led to the novel finding that genetic inheritance and shared environmental factors affecting leptin concentrations were over four-fold greater at the 90th than the 10th percentiles of the leptin distribution (Fig. 1). Traditionally, the decision to log transform data is driven solely by the statistical requirements of parametric testing. With respect to analyzing genotype–phenotype associations, the logarithmic and other normalizing transformations of right-skewed data accentuates the contribution of lower phenotypic values while diminishing the contribution of higher values. The logarithmic transformation eliminated the increase in β_FS_ with increasing percentiles of the leptin distribution and the greater β_FS_ in women than men, consistent with the conclusion that the genetic effects are concentration dependent. However, we are not aware of any biological rationale for analyzing normally distributed blood proteins as plasma concentrations and asymmetrically distributed blood proteins as log-concentrations. In fact, the majority of studies reporting gene-environment interactions involve untransformed leptin concentrations^42,43,45–47,49,58–60^.

Women have higher leptin concentrations than men due to their female body fat distribution and/or low testosterone^35–37^. The goal of sex-adjustment is to eliminate the male–female difference, usually through a translational adjustment of their respective distributions, to ideally attain comparability at each percentile of their respective distributions. Figure 1 suggests that the higher leptin concentrations in women than men should result in stronger female inherited or shared environmental effects on their leptin concentrations, as observed in Fig. 5. This resulted in a significant sex-difference between male and female β_FS_ when their age-and sex-adjusted data were matched at their corresponding percentiles (Fig. 5A), but not when matched by leptin concentrations (Fig. 5B), or when their leptin concentrations were analyzed without adjusting for sex (Fig. 5C). Rather than postulating sex-specific genetic effects^39^, we propose that the greater female than male leptin heritability reported by others^11,13,14,39,40^ may be entirely attributable to the women’s higher leptin concentrations.

Figures 6 and 7 display gene-environment and gene-disease interactions reported by others, where the larger genetic effect size of directly measures SNPs are associated with higher mean leptin concentration. They are examples where the authors’ various genetic speculations might be more simply explained by a single underlying phenomenon: quantile-dependent expressivity. None of the studies cited the differences in mean concentrations by environmental or disease condition as an explanation for the reported interactions.

### Caveats and limitations

An important limitation of our analysis is that β_FS_ does not only measure heritability (i.e. the proportion of the phenotype variance due to additive genetic effects). Falconer’s formula equate β_FS_ to (0.5V_A_ + 0.25V_D_ + V_Ec_)/V_P_ where V_A_ is the additive genetic variance, V_D_ the dominance variance, V_Ec_ the common environment variance, and V_P_ the phenotype variance^57^. Although there is no way to separate V_A_, V_D_, and V_Ec_ or to correct for assortative mating in our analyses, 2β_FS_ (i.e., 0.34 ± 0.04) is smaller than 75% of the heritability estimates published by others^8–19^, suggesting that V_D_, V_Ec_, and assortative mating effects are modest, and the observed quantile-effects are largely genetic. Other studies, in fact suggest spouse concordance and shared environmental effects are modest. For example, Hasselbach et al.^13^ identified no significant shared environmental effect. The lack of common familial environmental influence and spousal effects were also reported by Rotimi et al.^14^. Liu et al.^12^ did not identify any significant spousal effect, and attributed only 12% of leptin variance to the shared sibling environment.

None of the SNPs identified to date explain any more than a few percent of leptin or soluble leptin receptor heritability^22,23^, which means that the effects of any particular SNP is not necessarily constrained by results of Fig. 1. Not all studies show an increase in genetic effect size with increasing leptin concentrations, e.g., the C/T exon 6 PPARγ polymorphism had the same effect in obese women and men despite the women’s two-fold greater leptin concentrations^44^. Our analyses were derived from an exclusively White population which may not apply to other racial groups, e.g., Luke et al.’s^61^ report that that lower leptin concentrations of Nigerians (6.4 ± 0.3 ng/ml) than Jamaicans (15.0 ± 0.7 ng/ml) or African Americans (18.8 ± 0.4 ng/ml) did not correspond to lower leptin heritability (*h*^*2*^: 0.38, 0.25, and 0.43, respectively).

### Conclusion

Our principle finding is that the full-sib regression slope increases with increasing percentiles of the sibs’ leptin concentrations, and that this increase accelerates dramatically at higher portions of its distribution. Included in the regression slope are genetic effects, which on the basis of other heritability studies, we presume to be substantial. This suggests the expressivity of leptin concentrations is quantile-dependent, that quantile-dependent expressivity likely explains the larger genetic effects on women’s than men’s leptin concentrations, and may contribute to many purported gene-environment interactions affecting leptin. In seeking genetic variants affecting leptin and other traits, it may not make sense to accentuate the weaker genetic effects at the lower phenotype values while de-emphsizing the stronger genetic effects at the higher phenotype values.

## Supplementary information

Supplementary Information 1.

## Data Availability

The data used in these analyses are available data directly from the National Institutes of Health at https://biolincc.nhlbi.nih.gov/studies/framcohort/, https://biolincc.nhlbi.nih.gov/studies/gen3/ and https://biolincc.nhlbi.nih.gov/studies/framoffspring/ with requestor’s full or expedited IRB review.
